# Characterization of Red Wine Proanthocyanidins Using a Putative Proanthocyanidin Database, Amide Hydrophilic Interaction Liquid Chromatography (HILIC), and Time-of-Flight Mass Spectrometry

**DOI:** 10.3390/molecules23102687

**Published:** 2018-10-18

**Authors:** Scott Frost, Larry A. Lerno, Jerry Zweigenbaum, Hildegarde Heymann, Susan E. Ebeler

**Affiliations:** 1 Department of Viticulture & Enology, University of California, Davis, One Shields Avenue, Davis, CA 95616, USA; scfrost@ucdavis.edu (S.F.); lalerno@ucdavis.edu (L.A.L.); hheymann@ucdavis.edu (H.H.); 2 Agilent Technologies, Inc., Wilmington, DE 19808, USA; j_zweigenbaum@agilent.com

**Keywords:** proanthocyanidins, time-of-flight mass spectrometry, wine, ultrahigh performance liquid chromatography, hydrophilic interaction liquid chromatography, tandem mass spectrometry

## Abstract

Proanthocyanidins are complex polymers of flavan-3-ol monomers and play a key sensory and health role in foods and beverages. We describe here a novel method for characterizing wine proanthocyanidins using a theoretical database comprised of the chemical formula and exact mass of 996 compounds. The database was constructed using the four primary grape and wine proanthocyanidin monomers: (epi)catechin, (epi)catechin-3-*O*-gallate, (epi)gallocatechin, and (epi)gallocatechin-3-*O*-gallate, each combined in all possible combinations up to a polymerization of 10. The database was queried against spectra collected using ultrahigh performance liquid chromatography (UHLPC) with a hydrophilic interaction liquid chromatography (HILIC) column and coupled to a high-resolution accurate mass quadrupole time-of-flight mass spectrometer (Q-TOF MS). Two wine samples produced with different post fermentation maceration were analyzed using the presented method to demonstrate application for analysis of diverse proanthocyanidins. The first sample was pressed immediately at the end of fermentation when all sugar had been utilized and the second received eight weeks of post fermentation maceration. The HILIC column combined with high resolution tandem mass spectrometry and database matching provided tentative identification of 89 compounds with excellent resolution and without the need for two-dimensional separations. The identified compounds were visualized with Kendrick mass analysis, a simple technique allowing for rapid visualization of which compounds are present in a given sample.

## 1. Introduction

**Figure 1:**
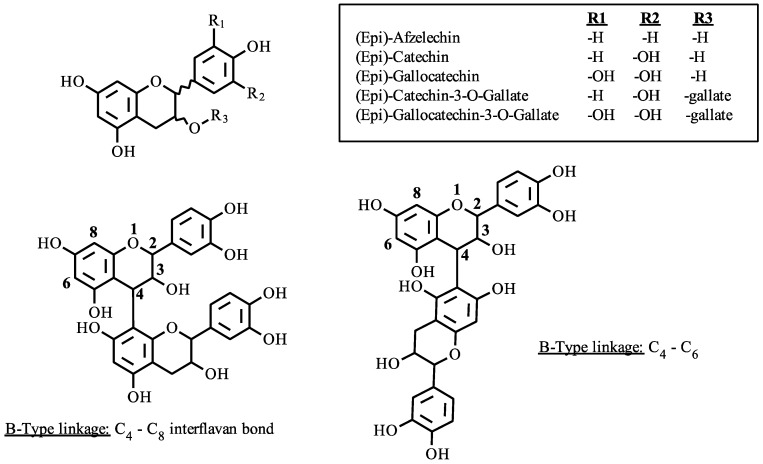
Proanthocyanidin linkages and polymer subunits.

Proanthocyanidins, or condensed tannins, are plant secondary metabolites produced by the polymerization of flavan-3-ol monomers. The monomer units are produced in the plant cell chloroplast but condensed in specialized chloroplast derived organelles termed tannosomes [1]. Proanthocyanidins are compositionally and structurally complex due to a variety of possible subunits and interflavan bond locations. Figure 1 displays the basic structure of a flavan-3-ol monomer with two illustrated interflavonoid linkages of single (B-type) linkage with either 4–8 or 4–6 bonds.

Given the variety of possible subunits, the number of potential compounds of a given polymerization can be expressed exponentially as *A^n^*, where *A* is the number of possible subunits and *n* is the polymer length. For example, a four-unit polymer comprised of the monomers catechin and gallocatechin, linked together through a C_4_–C_8_ interflavan bond (B-type) would give 16 possible compounds. Each isomer will have a different primary structure and molecular geometry. This variability has been shown to alter the colloidal state preference and protein binding affinity of proanthocyanidins [2]. This demonstrates the importance of proanthocyanidin structure on the molecular assembly, which gives rise to the astringent properties in foods and beverages.

As a result of the structural and chemical diversity, proanthocyanidins are challenging to measure. Often, bulk phenolic measures are collected using benchtop methods. For example, total phenol content can be measured using Folin-Ciocalteau or UV absorbance, while protein precipitation with bovine serum albumin (BSA) provides a quantification of the precipitable and non-precipitable fractions of a given sample [3]. Although relevant, benchtop methods do not provide compound specificity, such as degree of polymerization, molecular size, or monomer composition. Gel permeation chromatography (GPC) has been used to estimate molecular size [4], and monomer composition can be estimated by acid catalysis in the presence of excess phloroglucinol [5]. Each method is applied after proanthocyanidin isolation and fractionation (e.g., Sephadex LH-20). Thus, results are driven by the purity of a given fraction.

However, individual low molecular weight proanthocyanidin polymers have been analyzed using chromatographic separation followed by mass spectrometry. Reverse phase, C18 columns are frequently employed, but sufficient resolution of polymers larger than a degree of polymerization (DP) of four is rarely achieved [6]. In reversed phase separation the resulting chromatogram shows a pronounced increase in baseline during each analysis run, which has been shown to be unresolved higher DP compounds eluting during the course of the separation, making both quantitation and profiling of proanthocyanidins difficult.

Normal phase (NP) and hydrophilic interaction liquid chromatography (HILIC) methods have shown the ability to resolve higher DP proanthocyanidins. In comparing the methods under identical conditions, HILIC shows greater retention and thus increased resolution of isomers within a given degree of polymerization [7,8]. Additionally, the HILIC solvent system is compatible with reversed-phase (RP) which has led to the development of two-dimensional (2D) chromatographic methods. The enhanced resolving power of HILIC × RP has been utilized to identify proanthocyanidins in both wine and grape seeds [9,10]. Off-line 2D methods, requiring the collection of fractions in the first dimension, entails substantial time to process a single sample, and are not easily applied to large experiments or routine analysis. On-line methods, as compared to their off-line counterparts, require specialized equipment, frequently making this approach cost prohibitive.

Thus, additional methodologies have been employed to improve the measurement of proanthocyanidins. High resolution mass spectrometry (HRMS) is a powerful tool for the qualitative analysis of unknown compounds [11,12]. HRMS provides an accurate mass measurement allowing for the prediction of the molecular formula of unknown compounds. The predicted chemical formula, along with the accurate mass can then be used to characterize the various compounds of a complex mixture. This particular approach is well-suited for processing data collected from non-targeted mass spectrometry methods, providing what can be considered as the chemical fingerprint of a sample. The fingerprints of multiple samples can then be compared through multivariate statistics and van Krevelen visualization to evaluate differences within a sample set. This approach has been utilized to profile the chemical complexity of wine. In one such study, high-field Fourier transform ion cyclotron resonance mass spectrometry (FT-ICR MS) was used to gather the chemical fingerprint of several barrel-aged wines. The fingerprint of each wine was displayed by Van Krevelen visualization and showed the distributions of multiple compound classes. Ultimately, the power of this approach was shown in the statistical results, as the forest location where the barrel oak was grown could be discriminated using multivariate statistics (i.e., Partial Least Squares-Discriminant Analysis, PLS-DA) [13].

HRMS data can be further processed to identify specific members of a compound class through mass defect analysis [14]. The initial method was based on the conversion to a mass scale where the CH_2_ radical is set to 14.0000 mass units. Thus, compounds that differ by units of CH_2_ will have an equivalent mass defect, allowing homologues series in a complex sample mixture to be identified. This targeted filtering of HRMS data was first utilized to identify a series of alkyl naphthalene compounds. The increased availability of HRMS instrumentation has allowed these data analysis techniques to be used in broad range of fields, such as bovine milk lipids [15], lignin degradation products [16], and epicatechin-acetaldehyde condensation products [17].

The presented study reports a novel method for characterization of wine proanthocyanidins. A theoretical exact mass database was first constructed using the flavan-3-ol monomers found in grapes: (epi)catechin, (epi)catechin-3-*O*-gallate, (epi)gallocatechin, and (epi)gallocatechin-3-*O*-gallate. Chemical formulas and theoretical exact masses were calculated for all possible polymers up to DP 10, which returned a total of 996 compounds. Proanthocyanidins were extracted from a wine sample using an Amberlite resin, then analyzed using HILIC ultrahigh performance liquid chromatography-quadrupole time-of-flight (UHPLC-Q-TOF) HRMS. The collected mass spectrometry time-of-flight (MS TOF) spectra were searched against the database followed by integration of the returned chromatographic peaks. Identification of matched compounds was then tentatively confirmed using high resolution tandem mass spectrometry. A modified Kendrick mass defect analysis was utilized to visualize the isomeric relationships of identified compounds and rapidly identify the composition of each identified polymer. The method is demonstrated on two wine samples which vary by length of post fermentation skin contact time [18].

## 2. Results and Discussion

### 2.1. Initial Compound Identification: Agilent Profinder

A set of 996 theoretical polymers comprised of four flavan-3-ol subunits (catechin, C_15_H_14_O_6_; gallocatechin, C_15_H_14_O_7_; catechin-3-*O*-gallate, C_22_H_18_O_10_; and gallocatechin-3-*O*-gallate, C_22_H_18_O_11_) were utilized as query targets for molecular formula matching. In each case, the epimer is included as it has an identical chemical formula. This method was applied to each wine sample, extended maceration Em0 and Em8. Table 1 displays the retention times, chemical formula, polymer composition, and mean experimental mass for 89 compounds tentatively identified in the wine sample through molecular formula matching. Each of the 89 compounds were shown to have a unique retention time/chemical formula pairing, but overall a total of 21 different chemical formulas matching the personal compound database (PCD) were found within the collected Q-TOF MS spectra. For each mass, multiple isomers existed and these isomers were resolved chromatographically. These results demonstrate the complexity of proanthocyanidin analysis.

### 2.2. Chromatography

**Figure 2:**
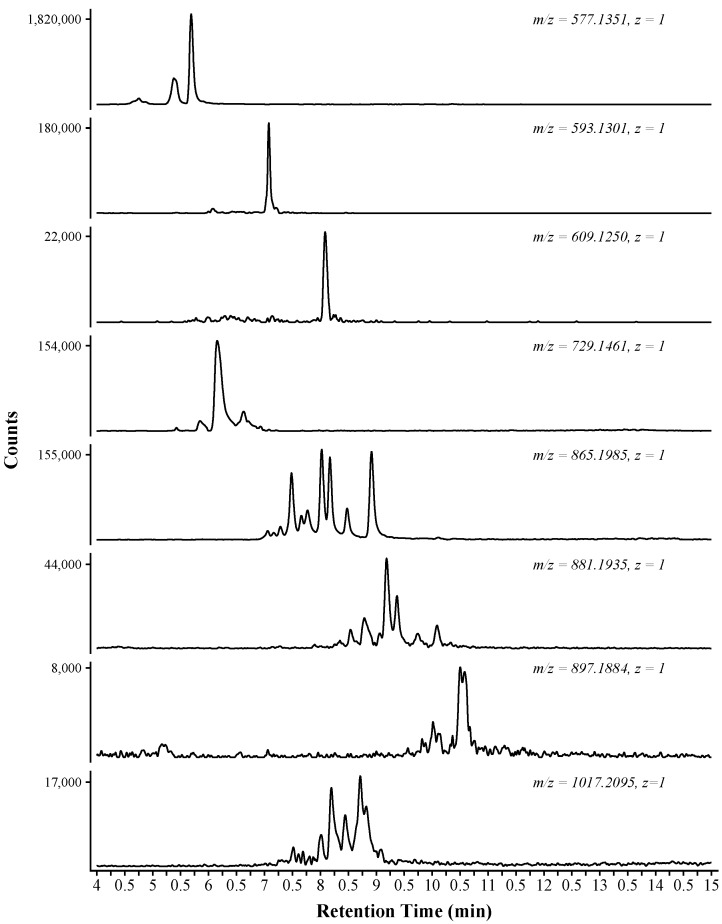
Selected Extracted Ion Chromatograms (EIC) for the eight-week maceration sample (Em8). The extracted mass and corresponding charge are indicated above each trace.

The amide HILIC column provided for the chromatographic separation of isomeric proanthocyanidins. Figure 2 displays extracted ion chromatograms from a subset of the identified compounds. The proanthocyanidins eluted during the time span of 4.5–16 min. The compounds that eluted prior to 4.5 min were monomeric phenolics, comprised mostly of small phenolic compounds and monomeric flavonoids (data not shown). The elution of the monomeric phenolics prior to the proanthocyanidins helps to reduce ion suppression of the proanthocyanidins that can occur if there is coelution with the more abundant monomeric phenolics [19]. The catechin-catechin dimers represent the largest peaks in the chromatograms, shown at 5.25–6 min. At 7 min, the catechin-gallocatechin peak represents the second most abundant proanthocyanidin by peak area.

Each extracted ion chromatogram trace is labeled with the detected *m*/*z* and corresponding charge (Figure 2). The first four panels were tentatively identified as dimeric proanthocyanidins, with the first panel showing the extracted ion chromatograms at *m*/*z* 577.1351. This extracted mass was identified as having a chemical formula of C_30_H_26_O_12_, a dimer comprised of catechin and epicatechin subunits. Multiple peaks are shown in the extracted ion chromatograms, with retention times of 4.68, 4.72, 5.31, and 5.63 min. Each peak corresponds to an isomer of C_30_H_26_O_12_. In total, four possible dimeric compounds exist, comprised of catechin and epicatechin units. In the presented example, the four peaks are shown, but two peaks are closely retained (4.68 and 4.72 min). Due to the chemical isomorphism, these similarly retained compounds are likely catechin-epicatechin and epicatechin-catechin dimers.

The resolving power of the amide HILIC column is best demonstrated by the proanthocyanidin trimer. Ten peaks were identified as the simple catechin/epicatechin trimer (Figure 2, 865.1985, *z* = 1; Table 1). These ten compounds can be accounted for by considering the number of possible trimer isomers with a formula of C_45_H_38_O_18_, each comprised of catechin and epicatechin subunits. Eight possible isomers exist (possible isomers = *A^n^*, *A* = polymerization, *n* = possible subunits) with each showing an identical interflavonoid linkage (e.g., C_4_–C_8_). The ninth and tenth compounds are tentatively identified as different linkage pattern (e.g., B-type; C_4_–C_6_). Previous methods have not reported resolution of the proanthocyanidin trimer to this degree. In an online 2D approach (diol HILIC × R-C18), one group has reported four trimer peaks at [M − H]^−^ 865.5 [20].

In addition to retention time shifts resulting from differences in catechin and epicatechin subunits, the inclusion of gallocatechin and/or a gallate unit also shifted the retention time. The inclusion of a gallocatechin subunit can be considered equivalent to the addition of an oxygen to the polymer, as the gallocatechin is defined by the trihydroxylated B-ring. The inclusion of gallocatechin resulted in an increased retention time as the polymer shows increased hydrophilicity and a stronger interaction with the stationary phase [21]. This can be seen by evaluating the mean retention time of compounds with equivalent polymerization but different subunit composition. The base (epi)catechin trimer group had a mean retention time of 7.82 min (Table 1). The addition of an oxygen to the polymer, e.g., comparing Cat-Cat-Cat to Cat-Cat-GalCat, shifted the mean retention time by approximately one minute to 8.78 min. This trend continues as Cat-GalCat-GalCat group has a mean retention time of 9.51 min. The addition of gallate to the polymer shows a similar retention behavior, but the shift is not as large as the addition of a hydroxyl group. This can be contrasted with the addition of gallic acid to the polymer, which also increases retention and overall polymer polarity, but not to the extent of the hydroxyl addition. This difference is likely due to the reduced polarity of the overall molecule resulting from the ester functional group.

Compound retention within the HILIC system increases as a result of an overall increase in hydrophilicity. In the acidified solvent system, each hydroxyl group is charged, creating a stronger interaction between the analyte and the water/methanol aqueous phase. It has been shown that a portion of the aqueous phase is immobilized within the amide stationary phase [22]. This allows for liquid-liquid partitioning based on the difference in polarity between the bulk phase and the immobilized aqueous phase. As the water percentage increases in the bulk phase, the polarity difference between the two decreases allowing for elution to occur [23]. Interaction between the analyte and the stationary phase also drives retention differences [24]. Electrostatic interactions and hydrogen bonding will increase retention.

As polymerization increases, the number of possible isomers for a given compound also increases. In the presented dataset, the highest polymerization identified was a single peak of DP 7 (Table 1). Higher polymerization compounds were not identified here, but have been reported in previous HILIC methods, that analyzed raw unfermented cacao [25] and cranberries [26]. This observation may be a result of wine production conditions. Wine is an acidic environment with elevated temperatures, thus interflavonoid bond cleavage may have occurred resulting in a shift to smaller compounds. It is also possible that the lager compounds were adsorbed to the pomace and lees, then removed at pressing [27,28,29,30,31] and thus would not be observed in the wine samples analyzed.

The overall complexity of the wine samples could also result in undetected compounds. As the degree of polymerization increases, the number of possible compounds exponentially increases and the probability of a specific compound occurring decreases. Given the demonstrated resolution, higher polymerization would resolve into the baseline. In addition, difficulty in ionizing the large polymers could also limit detection of low abundance compounds.

### 2.3. MS/MS Identification of Putative Compounds

**Figure 3:**
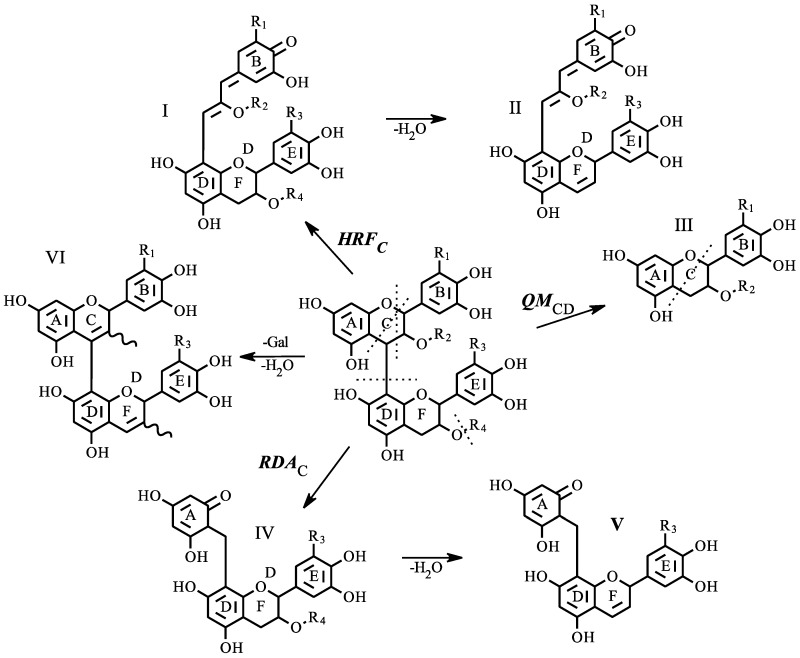
Characteristic proanthocyanidin fragmentation pathways. Three primary fragmentation mechanisms are shown: Heterocyclic Ring Fission (HRF), quinone methide fission (QM), and retro Diels-Alder (RDA); subscripts C or CD indicate the fragmentation location in the rings of the subunit; each yield structures I, III, and IV, as indicated. Further losses of water and gallate (II, V, VI) are also indicated. For each structure; R_1_ = R_3_ = OH, H: R_2_ = R_4_ = H, Gallate.

Compounds tentatively identified by database matching were further confirmed using tandem mass spectrometry. Three specific fragmentation mechanisms were shown to provide diagnostic ions allowing for confirmation. These mechanisms have been previously described for positive mode tandem mass spectrometry [30,31]. Figure 3 displays the three primary pathways for a generic proanthocyanidin dimer, with additional pathways indicating water and gallic acid loss. Structure I was produced from heterocyclic ring fission of the flavonoid C-ring (HRF_C_), splitting occurs between the oxygen at position 1 and the carbon at position 2, then diagonally across the six-member ring between carbons 4 and 5. Phloroglucinol is produced as the neutral loss, and the remaining charged ion can be diagnostic as the B-ring substitution pattern is retained. The structure, I, can be further reduced by loss of gallate and water to structure II (Figure 3).

The splitting of the interflavan bond results from quinone methide fission (QM_CD_). In Figure 3, this is shown as structure III. From a diagnostic standpoint, this fragmentation pattern will provide information on which flavan-3-ol subunits are present in the structure. From the collected spectra in the wines analyzed here, three different flavan-3-ol units were found. The catechin unit was identified with two different *m*/*z* values (289.0781, 287.0561) depending on the original position within the polymer; *m*/*z* 287.0561 represents an extension unit. (Epi)catechin-3-*O*-gallate and (epi)gallocatechin also returned a signal (*m*/*z* 441.0827, 305.0667), but in contrast to catechin, each was only measured as terminal units. The retro Diels-Alder (RDA_C_) reaction is also a characteristic mechanism of flavonoid tandem mass spectrometry. The cleavage product retains a complete flavonoid structure along with the A-ring of an upper flavonoid unit via the interflavan bond (structure IV, Figure 3). The dehydrated form of the breakdown product was the commonly measured component (structure V) in the wines analyzed in this study. In many cases, structure V was the base peak within the tandem spectra.

**Figure 4:**
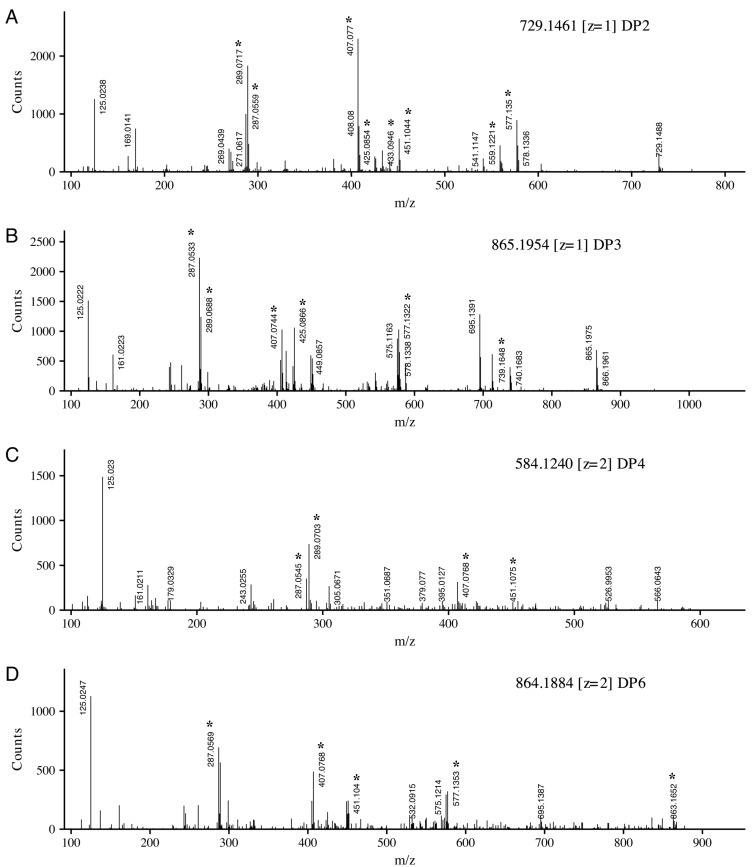
Negative ion electrospray ionization (ESI) tandem mass spectra of four representative proanthocyanidins. Precursor ion and charge are indicated. Diagnostic ions associated with cleavage patterns described in Figure 3 are indicated with *. Degree of polymerization (DP).

Using this set of diagnostic structures and the B-ring substitution patterns, compounds identified through database matching can be further confirmed. Figure 4 displays four collected sample tandem mass spectra, each spectrum is labeled with the precursor ion and charge. Panel A was tentatively identified as a dimer of (epi)catechin and (epi)catechin-3-*O*-galate with unfragmented precursor ions observed at *m*/*z* 729.1488. The fragment at *m*/*z* 577.1350 was identified as the charged (epi)catechin-(epi)catechin dimer. The difference between these ions can be accounted for by the loss of gallate from the precursor. Additionally, *m*/*z* 577.1350 is represented by structure VI (R_1_ = R_3_ = H) in Figure 3, with loss of water yielding *m*/*z* 559.1221. From the precursor ion, HRF_C_ yields *m*/*z* 451.1044 and *m*/*z* 433.0946 with water loss, each representing structures I and II (R_1_ = R_3_ = H), respectively. Continuing to utilize Figure 3, RDA_C_ leaves structure IV (*m*/*z* 425.0854, R_1_ = R_3_ = H) and structure V (*m*/*z* 407.0770) with loss of water. The fragments at *m*/*z* 289.0717 and *m*/*z* 287.0559 represent the flavonoid units resulting from QM_CD_. The lack of an ion at *m*/*z* 305.0667 is key, as its absence signifies that (epi)gallocatechin is not an extension unit, providing further confirmation that the collected spectra is a dimer of (epi)catechin and (epi)catechin-3-*O*-gallate.

Similar fragment identification strategies can also be applied to the collected tandem spectra in Figure 4B–D. Figure 4B displays a catechin trimer with precursor ion *m*/*z* 865.1954. In addition to similar fragmentation patterns shown in Figure 4A, two additional diagnostic ions are shown. The first ion measured at *m*/*z* 739.1648 is equivalent to structure I (R_1_ = R_2_ = R_3_ = H) with an additional catechin. The second key fragment at *m*/*z* 695.1319 is structure VI (R_3_ = R_4_ = H) with an additional (epi)catechin. These two ions fragments, in addition to the absence of the (epi)gallocatechin ion (*m*/*z* 305.0667), provide putative confirmation of this spectrum as the catechin trimer.

Figure 4C,D display the spectra of doubly charged (*z* = 2) precursor ions. The tetramer of three catechin units and a single gallocatechin is displayed. Fragments at *m*/*z* 289.0703 and *m*/*z* 305.0671 indicate catechin and gallocatechin extension units. Figure 4D displays the collected spectra of a catechin hexamer (*m*/*z*, *z* = 2 864.1884), showing a similar pattern as that observed in the smaller catechin polymers, with the addition of a fragment at *m*/*z* 863.1652. This ion fragment is an extension section comprised of a three catechin subunit.

### 2.4. Analysis of Wine Samples

Two wine samples, processed by different techniques, were used to demonstrate the application of this method to the analysis of the distribution of proanthocyanidins in wine. These samples were part of a broad study evaluating the effect of cap management and extended maceration on chemical and sensory measures of red wine [18,32]. The two wines were produced using identical fermentation conditions; at the end of the primary alcoholic fermentation one sample was immediately pressed off the skins (Em0) while for the second sample, the wine was allowed to remain in contact with the pomace (grape skins and seeds) for eight weeks post-fermentation (Em8). This production practice, called extended maceration, is commonly applied in commercial wineries, and provides a winemaking tool for altering the astringency, bitterness, and body of red wine.

HILIC chromatography in conjunction with Electrospray Ionization (ESI)-Q-TOF MS was utilized to capture mass spectral data followed by post processing targeted mass filtration as described above to identify compounds of interest within the two wines. Given the possible complexity of proanthocyanidins found in a wine, the ability to quickly profile which compounds are present or absent is important in determining the effect on the sensory properties of the wine. Kendrick mass analysis allows for rapid visualization of which compounds were identified in a sample.

**Figure 5:**
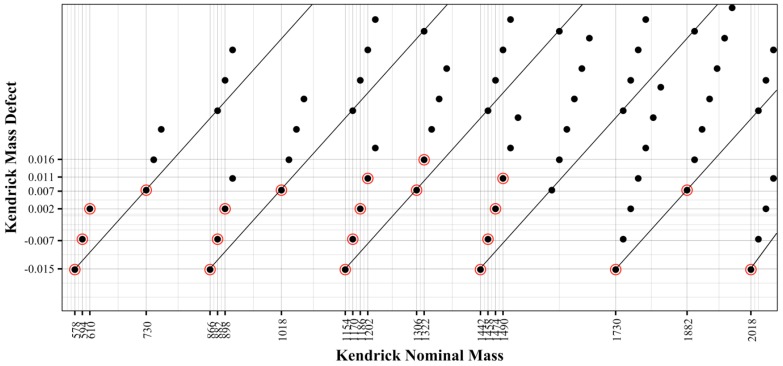
Kendrick defect analysis, created by plotting the Kendrick mass defect (KMD) and Kendrick nominal mass (KNM). Each solid black point represents the KNM and KMD of the proanthocyanidin calculated in the theoretical database. Compounds which were identified in the wine samples are encircled in red.

Kendrick mass analysis was applied to visualize the specific identified compounds. The presented approach uses the simple (epi)catechin extension unit (C_15_H_12_O_6_) as the basis of the Kendrick mass scale, and information can be obtained regarding subunit composition. This was done by first plotting the Kendrick Mass Defect (KMD), Equation (2), against the Kendrick Nominal Mass (KNM), Equation (1), for each compound. Figure 5 displays each of the theoretical polymers as solid black dots, along with red circles indicating a putatively identified compound found in the wine samples. The x- and y-axes are labeled with the KNM and KMD for the identified compounds. This visualization allows one to quickly determine which polymers are present in a sample. The diagonal lines divide the graph by degree of polymerization, e.g., the nine points in the far-left diagonal lane of the plot are the nine possible dimers. The next lane to the right shows the trimers. Evaluating the KNM of 866, the simple catechin-catechin-catechin trimer is positioned with a defect of −0.015. This defect is characteristic of a polymer comprised of catechin repeats and is also shown by the catechin tetramer (1154 KNM), pentamer (1442 KNM), and the hexamer (1730 KNM) as each are plotted with an equivalent −0.015 mass defect.

Addition of gallic acid and hydroxylation of the base polymers further impart complexity into the Kendrick analysis. Evaluating within a single degree polymerization, the addition of gallic acid or hydroxylation to the base (epi)catechin polymer produces a specific pattern within the Kendrick plot (Figure 5). For example, the trimer (866 KMD) shows groups of four compounds, as indicated along the diagonal line. From the simple catechin trimer (cat-cat-cat), if one catechin unit is replaced with gallocatechin (cat-cat-galcat), thus adding one additional oxygen to the chemical formula, the nominal mass increases from 866 to 882 and the defect increases from −0.0152 to −0.0066. The addition of a second gallocatechin subunit (cat-galcat-galcat) further shifts the defect and nominal mass by 16 and 0.009 respectively to 898 and 0.0020. Continuing with the basic catechin trimer (cat-cat-cat), an addition of gallic acid (cat-cat-cat:gal) will shift the nominal mass to 1018 and the defect to 0.0073. The addition of a second catechin gallate (cat-cat:gal-cat:gal) will further shift the nominal mass to 1170 and the defect to 0.0298. Overall, from any given polymer, the addition of an oxygen shifts the nominal mass by 16 and the defect by 0.0086. The addition of gallate to a polymer will shift the nominal mass by 152 and the defect by 0.0225.

**Figure 6:**
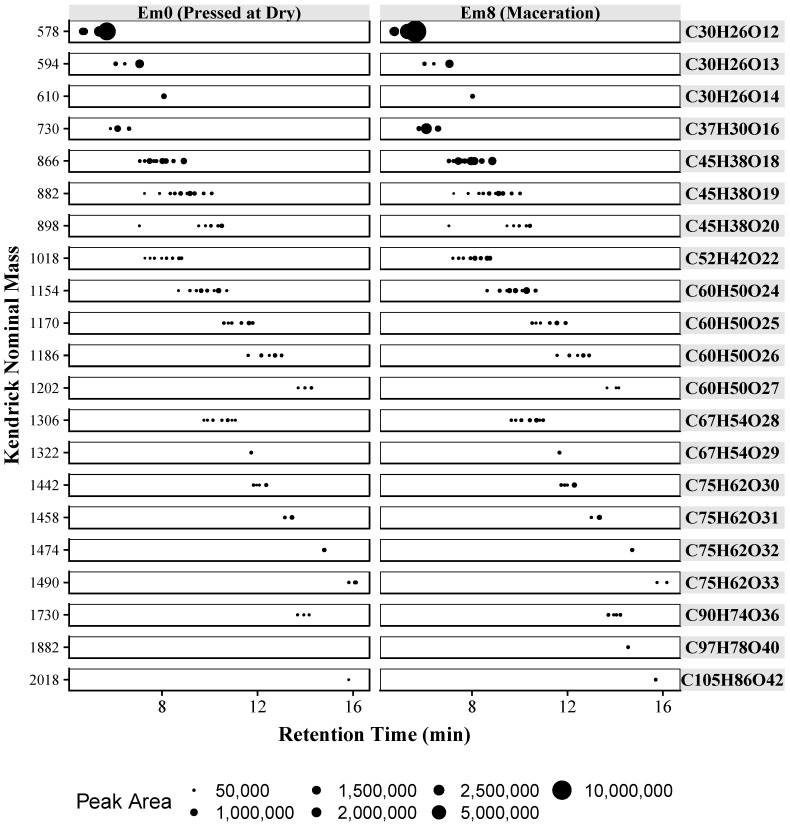
Proanthocyanidin polymers in Merlot wines produced with two different maceration techniques. Each dot represents a putative proanthocyanidin compound. Hydrophilic interaction liquid chromatography (HILIC) retention time is plotted vs. Kendrick nominal mass and absolute peak area is indicated by the dot size. Each horizontal lane indicates the nominal mass and the identified chemical formula. Extended maceration (Em).

In addition to Kendrick mass, chromatographic peak areas can be used to evaluate relative treatment effects. In Figure 6 each tentatively identified compound, by KNM, is plotted against retention time, with each point scaled to the absolute peak area. Twenty-one different formulas are shown, with a total of 89 tentatively identified compounds. By plotting the KNM and cross referencing to Figure 5 it is possible to determine the composition of a given polymer. Overall, Figure 6 provides a rapid visualization of the relative difference between the two samples. On visual inspection, the catechin/epicatechin dimer at KNM of 577 is the most abundant in each sample. In general, the sample with extended maceration (Em8) shows a higher concentration of each polymer. In comparing the two samples, longer skin contact (Em8) also resulted in higher concentrations of galloylated polymers, as demonstrated by comparing the relative peak areas of compounds with KNM 730 and 1018. An overall trend showing increased extraction of proanthocyanidins with maceration is shown. Both the increase in smaller DP proanthocyanidins and the greater abundance of galloylated proanthocyanidins suggests increased proanthocyanidin extraction from the seeds. Prior research has shown that seed proanthocyanidins have smaller DPs and are more highly galloylated than skin derived proanthocyanidins [33]. Similar findings regarding increased seed extraction have been reported for Merlot wines, in which an increase of approximately 73% in seed extraction was observed with a 30 days extended maceration [34].

The method described here provided information on the abundance of specific grape derived proanthocyanidins in the wine samples. Using Q-TOF MS, each proanthocyanidin was directly detected and putative structures assigned based on MS/MS fragmentation patterns. This is in contrast to alternative methods that require acid cleavage or measurement of a bulk property of proanthocyanidins (e.g., protein precipitation, Folin-Ciocalteau) for detection and structural identification.

## 3. Materials and Methods

### 3.1. Chemical and Reagents

MS grade acetonitrile, methanol and formic acid (>99.5%) were purchased from Fisher Scientific (Fair Lawn, NJ, USA); ammonium formate and XAD7HP Amberlite resin were purchased from Sigma-Aldrich (St. Louis, MO, USA). An in-house water purification system was used to produce 18 MΩ water for all analytical solutions and mobile phases.

### 3.2. Wine Production

Two red wine samples from an experiment evaluating the impact of specific wine production practices on sensory and chemistry were utilized [18,32]. Detailed wine production is described [18], but a brief description follows. Wines were made in the UC Davis Experimental Winery in Fall 2013. *Vitis vinifera* cv. Merlot with a total soluble solids measurement of 27.4 Brix, was machine harvested from the UC Davis Oakville Research Station (Napa, CA, USA). Fruit was crushed, destemmed, and pumped into six Cypress/UC Davis research fermentors, as described in Lerno [35]. All fermentors were inoculated 24 h after crushing and fermentation began within 72 h. During alcoholic fermentation, pump-overs were applied three times daily by a built-in fermentor pump and one tank volume was cycled for each pump-over. When the measured Brix had decreased to 14 °C, malolactic fermentation (MLF) was initiated. At the completion of alcoholic fermentation, three fermentors were pressed using a hydraulic press. The wine in the second set of three fermentors underwent extended maceration for eight weeks, during which the wines remained in contact with the pomace. During this maceration period, each fermentation tank was pumped over for five minutes per day and cap temperatures were maintained at 22 °C. At completion of MLF, wines were bottled and stored for analysis. The wine sample pressed at the completion of fermentation will be indicated as Em0, and the extended maceration sample is indicated as Em8.

### 3.3. Sample Preparation

Wine proanthocyanidins were extracted by rotary evaporating 250 mL of wine under reduced pressure at 35 °C to remove ethanol. Once the ethanol was removed the aqueous sample was loaded onto a XAD7HP (Sigma-Aldrich, St. Louis, MO, USA) column, followed by washing with four bed volumes of 0.1% aqueous formic acid. The bulk proanthocyanidin extract was eluted with 80% acetone/0.1% formic acid, followed by rotary evaporation under reduced pressure at 35 °C to remove acetone. The aqueous proanthocyanidin extract was then lyophilized to remove water. Lyophilized extract was then resuspended at a concentration of 0.5% (*m*/*v*) in 60% acetonitrile/20% methanol/20% water followed by filtration through 0.45 μm polytetrafluorethylene (PFTE) syringe filter into amber, glass 1.5 mL HPLC sample vials.

### 3.4. UHPLC-Q-TOF MS Analysis

All wines were analyzed in triplicate using an Agilent 1290 UHPLC coupled to an Agilent 6530 quadrupole time-of-flight mass spectrometer (Q-TOF MS). Instrument control and data acquisition was performed in Agilent MassHunter Acquisition (ver. 6). Prepared proanthocyanidin samples were injected (5 μL) onto an Agilent AdvanceBio Glycan Mapping (2.1 × 150 nm, 1.8 μm) HILIC amide phase column protected by a guard column of the same phase. The mobile phases were (A) 95% acetonitrile: 5% water with 10 mM ammonium formate and 0.2% formic acid; (B) 47.5% methanol: 52.5% water with 10 mM ammonium formate and 0.2% formic acid. The following solvent gradient was employed: 0 min, 0% B; 23 min, 50% B; 25 min, 100% B; 29 min, 100% B. The flow rate (0.4 mL min^−1^), column compartment (50 °C) and thermostatted autosampler were maintained for the duration of each analysis. Samples were analyzed in negative mode using an Agilent Dual ESI Jet Stream source. Nitrogen was used for both drying and sheath gas. Capillary voltage was set to 4500 V, fragmentor voltage 155 V, nozzle voltage 2000 V, drying gas at 10 L min^−1^ at 350 °C, sheath gas at 12 L min^−1^ at 350 °C, and the nebulizer was set to 25 psig. Spectral data were collected from 100 *m*/*z* to 3200 *m*/*z* at an acquisition speed of 3 spectra s^−1^. The Q-TOF MS was externally calibrated daily prior to analysis start, and reference mass correction was utilized to ensure mass accuracy. Tandem MS spectra were collected using identical chromatographic and source conditions, with an acquisition rate of 5 spectra s^−1^. A ramped collision energy was applied with slope 1.75× (precursor *m*/*z* /100) and offset ±15 eV. A preferred list of ions was utilized to target specific ions for tandem spectra collection.

### 3.5. Data Analysis and Workflow

For the theoretical proanthocyanidins database, four base subunits: Catechin (C_15_H_14_O_6_, “Cat”), gallocatechin (C_15_H_14_O_7_, “GalCat”), catechin-3-*O*-gallate (C_22_H_18_O_10_, “Cat:Gal”) and gallocatechin-3-*O*-gallate (C_22_H_18_O_11_, “GalCat:Gal”), were utilized to create a theoretical proanthocyanidin database of monoisotopic mass and chemical formulas. These four subunits represent the primary grape derived subunits. In each case, the corresponding epimer (e.g., catechin vs. epicatechin) is also possible; the identical theoretical mass would be calculated. Theoretical oligomers from all possible combinations of the four units from a polymerization of 2 up to 10 were calculated. This returned a total of 996 possible polymers, which were then contained as a personal compound database (PCD) within the Agilent software suite.

For the Profinder feature extraction: Identifying experimental masses, collected Q-TOF MS spectra were analyzed in Agilent Profinder (ver. 8). The find by formula and database matching were applied using the calculated theoretical proanthocyanidin database as the target. Initial mass extraction was set to exclude signal smaller than 750 counts. Negatively charged signal comprised from deprotonation and/or formate adducts, a charge state of −1 or −2, following the common isotope model were extracted. The extracted feature list was then queried against the theoretical proanthocyanidin data base set to a ±20 ppm tolerance. Kendrick calculations are as follows:

The converted mass scale was based on the simplest polymer extension, catechin less 2 hydrogens (C_15_H_12_O_6_). Conversion to this scale was achieved through applying Equation (1).

(1) $$Kendrick\;mass=IUPAC\;mass\;\times \;\frac{288}{288.0634}$$

The results of Equation (1) were then rounded to the nearest integer giving the Kendrick nominal mass. The Kendrick mass defect is then calculated by subtracting the Kendrick mass from the Kendrick nominal mass, Equation (2).

*Kendrickmassdefect* = (*nominalmass* ‒ *Kendrickmass*) (2)

These calculations were applied to each compound in the theoretical database and the matched experimental masses outputted using the Agilent Profinder algorithm.

## 4. Conclusions

A novel method was demonstrated to directly characterize proanthocyanidins by utilizing a targeted database to query collected spectra from an ultrahigh performance liquid chromatography system equipped with a hydrophilic interaction chromatography column coupled with high resolution Q-TOF MS. Using the described method, the targeted database search returned 89 putatively identified compounds from a total of 21 unique molecular formulas and differences in proanthocyanidin composition were observed as a function of skin contact time for two *V. vinifera* L. cv. Merlot wine samples. The approach does not require off- or on-line 2D separations to resolve the complex isomeric proanthocyanidin structures up to DP 7 observed in wines. The developed method is also faster than 2D separations and approaches such as phloroglucinolysis. As a result, it can be readily applied to analysis of large numbers of samples. The method presented is also applicable to the study of proanthocyanidins from other sources, such as white wine, with adjustments made to sample preparation to account for changes in proanthocyanidin content.

## Figures and Tables

**Table 1:** Retention times, chemical formulae and mean measured experimental mass of identified compounds. Polymer subunits are indicated as follows: (epi)catechin = Cat, (epi)catechin-3-*O*-gallate = Cat:Gal, (epi)gallocatechin = GalCat, and (epi)gallocatechin-3-*O*-gallate = GalCat:Gal.

Retention Time (min)	Mean Retention Time (min)	Formula	Polymer	Mean Experimental Mass ^1^	Mean Mass Error (ppm)
4.68, 4.72, 5.31, 5.63	5.09	C_30_H_26_O_12_	Cat-Cat	578.1401	4.0
6.01, 6.38, 7.05	6.48	C_30_H_26_O_13_	Cat-GalCat	594.1308	11.0
8.03	8.03	C_30_H_26_O_14_	GalCat-GalCat	610.1264	9.6
5.78, 6.09, 6.59	6.15	C_37_H_30_O_16_	Cat-Cat:Gal	730.1480	7.4
7.04, 7.24, 7.44, 7.61, 7.72, 7.97, 8.12, 8.42, 8.86	7.82	C_45_H_38_O_18_	Cat-Cat-Cat	866.2011	5.4
7.24, 7.85, 8.30, 8.49, 8.74, 9.01, 9.13, 9.32, 9.69, 10.04	8.78	C_45_H_38_O_19_	Cat-Cat-GalCat	882.1940	7.6
7.03, 9.49, 9.77, 10.00, 10.30, 10.46	9.51	C_45_H_38_O_20_	Cat-GalCat-GalCat	898.1860	10.7
7.22, 7.46, 7.65, 7.95, 8.15, 8.39, 8.66, 8.77	8.03	C_52_H_42_O_22_	Cat-Cat-Cat:Gal	1018.2106	6.1
8.65, 9.13, 9.41, 9.59, 9.85, 10.14, 10.32, 10.69	9.72	C_60_H_50_O_24_	Cat-Cat-Cat-Cat	1154.2613	6.8
10.54, 10.70, 10.89, 11.29, 11.58, 11.86	11.14	C_60_H_50_O_25_	Cat-Cat-Cat-GalCat ^2^	1170.2537	8.9
11.62, 12.10, 12.45, 12.69, 12.94	12.36	C_60_H_50_O_26_	Cat-Cat-GalCat-GalCat ^2^	1186.2434	13.2
13.69, 13.95, 14.20	13.95	C_60_H_50_O_27_	Cat-GalCat-GalCat-GalCat ^2^	1202.2393	12.2
9.70, 9.86, 10.09, 10.45, 10.72, 10.88, 11.02	10.31	C_67_H_54_O_28_	Cat-Cat-Cat-Cat:Gal ^2^	1306.2720	6.2
11.68	11.68	C_67_H_54_O_29_	Cat-Cat-Cat-GalCat:Gal ^2^	1322.2583	12.7
11.77, 11.91, 12.02, 12.31	12.00	C_75_H_62_O_30_	Cat-Cat-Cat-Cat-Cat ^2^	1442.3256	4.8
13.07, 13.38	13.23	C_75_H_62_O_31_	Cat-Cat-Cat-Cat-GalCat ^2^	1458.3216	4.0
14.75	14.75	C_75_H_62_O_32_	Cat-Cat-Cat-GalCat-GalCat ^2^	1474.3129	6.5
15.8, 16.06	15.93	C_75_H_62_O_33_	Cat-Cat-GalCat-GalCat-GalCat ^2^	1490.2938	15.8
13.66, 13.88, 14.09, 14.27	13.98	C_90_H_74_O_36_	Cat-Cat-Cat-Cat-Cat-Cat ^2^	1730.3864	5.5
14.6	14.60	C_97_H_78_O_40_	Cat-Cat-Cat-Cat-Cat-Cat:Gal ^2^	1882.3969	5.3
15.77	15.77	C_10_5H_86_O_42_	Cat-Cat-Cat-Cat-Cat-Cat-Cat ^2^	2018.4477	5.8

^1^ Mean measured mass of the molecule (not the ion), calculated over all samples analyzed; ^2^ Doubly charged species.
